# The Infection, Coinfection, and Abundance of Intestinal Protozoa Increase the Serum Levels of IFABP2 and TNF-α in Patients With Rheumatoid Arthritis

**DOI:** 10.3389/fmed.2022.846934

**Published:** 2022-04-12

**Authors:** Iris Paola Guzmán-Guzmán, Benjamín Nogueda-Torres, Oscar Zaragoza-García, José Eduardo Navarro-Zarza, Olivia Briceño, Gloria Pérez-Rubio, Ramcés Falfán-Valencia, Ilse Adriana Gutiérrez-Pérez, Isela Parra-Rojas

**Affiliations:** ^1^Faculty of Chemical-Biological Sciences, Universidad Autónoma de Guerrero, Chilpancingo, Mexico; ^2^Department of Parasitology, Escuela Nacional de Ciencias Biológicas, Instituto Politécnico Nacional, Mexico City, Mexico; ^3^Department of Internal Medicine, Hospital General Dr. Raymundo Abarca Alarcón, Chilpancingo, Mexico; ^4^Center for Research in Infectious Diseases, Instituto Nacional de Enfermedades Respiratorias Ismael Cosío Villegas, Mexico City, Mexico; ^5^HLA Laboratory, Instituto Nacional de Enfermedades Respiratorias Ismael Cosío Villegas, Mexico City, Mexico

**Keywords:** intestinal protozoa, infection status, IFABP2, inflammation, rheumatoid arthritis

## Abstract

Protozoa, nematodes, and platyhelminths are of clinical interest due to their role on the modulation of the immune responses. To determine the frequency of infection by intestinal parasites as well as the status of single or mixed infection (coinfection) and its relation with inflammation and intestinal permeability markers in patients with rheumatoid arthritis (RA), a cross-sectional study was conducted in 18 women diagnosed with RA. A fecal sample of each participant was analyzed for parasitic identification. The DAS28-erythrocyte sedimentation rate score, as well as the serum levels of TNF-α, IL-10, IL-17A, and the intestinal fatty-acid binding protein 2 (IFABP2), was determined through the ELISA technique. The T CD4+ and CD8+ lymphocytes' proportions were determined by flow cytometry. In this study, 50% (*n* = 9) of the total sample tested were positive to the presence of intestinal protozoa (27% by single infection and 22.2% by coinfection). *Blastocystis sp*. and *Endolimax nana* were the most frequently identified protozoa. The serum levels of IFABP2 were increased in patients with infection by protozoa, mainly in those individuals with coinfection and a larger abundance of *Blastocystis sp*. We found that coinfection by protozoa was related to higher levels of TNF-α and higher frequency of T CD4+ lymphocytes, mainly in patients under antirheumatic treatment. Infection by intestinal protozoa is associated with increased intestinal permeability in patients with RA; thus, infection, coinfection, and abundance of intestinal protozoa should be clinically screened because they could be an associated factor to the clinical variability of the disease.

## Introduction

Elements of the intestinal microbiota such as protozoa, nematodes, and platyhelminths are of great clinical interest due to their role in modulating the immune response and as potential triggers of the autoimmune process in rheumatoid arthritis (RA) (1–3). In the last few years, it has been revealed that intestinal dysbiosis has an important physiopathological function in the development of RA, as well as in the clinical manifestation of the disease and, potentially, in the response to antirheumatic treatment (4–7). Interestingly, it has been previously described that the composition of gut microbiota could influence the modulation and progression of parasitic infections (8), and at the same time, infection by intestinal parasites could promote intestinal dysbiosis (9, 10).

In patients with RA, several studies have reported that infections by protozoa (11–20) and by nematodes (16, 21, 22) are related to the development of inflammatory syndromes. The infection by enteric pathogens such as protozoa has an effect on the regulation of genes that are related to the stress response and cell proliferation, thus promoting the chemokines' synthesis and inflammation (23). On the other hand, some helminth products such as glycoprotein ES-62 decrease the damage of intestinal barrier in RA (24) and have anti-inflammatory effects by modulating the Th1 and Th17 immune responses (25), as well as the cell infiltration in joints, the reactivity of effector B cells, and the restoration of IL-10-producing B cells (26).

It has been demonstrated that the infection by parasites such as *Blastocystis sp*., *Giardia sp*., *Cryptosporidium sp*., *Trichuris sp*., *Entamoeba histolytica, Nippostrongylus brasiliensis*, and *Heligmosomoides polygyrus* could modulate the intrinsic and extrinsic apoptosis pathways in intestinal epithelial cells in the host (27), increasing the intestinal epithelial permeability and the bacterial translocation due to the loss of adherents junctions (E-cadherin and α-catenin) (28) and tight junctions [occludin, claudin 3, claudin 4, and zonula occludens-1 (ZO-1)] that maintain the enterocytes' paracellular integrity (28–32). Different molecules have been considered useful during the evaluation of intestinal permeability. ZO-1 has been considered an intestinal barrier functionality and integrity marker, mainly in the early stages of autoimmune diseases' development (33) and in the progression of chronic inflammatory disorders (34). Other markers such as D-dimer, a-glutathione S-transferase, and the intestinal fatty-acid binding protein 2 (IFABP2) have been related to the presence of intestinal mucosa's damage (35). IFABP2 is a 15-kDa cytosolic protein present in the epithelial cells of the small and large intestines, particularly in the duodenum, jejunum, ileum, and colon (36). IFABP2 function in the intestinal enterocytes is promoting their energetic homeostasis (37) and transporting long-chain fatty acids (38). It has been reported that the levels of expression of IFABP2 represents a marker for intestinal lesions (39, 40). Cascais-Figueiredo et al. (41) reported that the levels of IFABP2 are increased during *Giardiasis*, showing the potential relation between infections by protozoa with increased intestinal permeability. The present study has the objective to determine the frequency of intestinal parasitosis in its single or mixed infection status, and its relationship with serum levels of IFABP2 and inflammation markers in a group of women from southern Mexico who have been diagnosed with RA.

## Materials and Methods

### Patient's Selection

A cross-sectional pilot study was conducted on 18 women diagnosed with RA according to the 2010 American College of Rheumatology (ACR) and the European League Against Rheumatism (EULAR) criteria (42). They were diagnosed and treated at the Rheumatology Department of the General Hospital in Chilpancingo, Mexico. The recruitment of the patients was performed from March to November of 2019. All patients were surveyed to obtain sociodemographic data. Patients diagnosed with peptic ulcer and inflammatory bowel diseases were also excluded, as well as those with gastrointestinal surgeries (e.g., gastrectomy, bariatric surgery, and colectomy). Moreover, we considered as an exclusion criteria having had antiparasitic treatment at least 6 months before taking the sample. The clinical and treatment characteristics were evaluated during the consultation and from the clinical file. All patients agreed to participate and gave their informed consent in writing. This study was approved by the Ethics and Research Committee of the University Autonomous of Guerrero (Project identification code: CB-004/2017).

Patients with an overlapping syndrome (characteristics of two autoimmune connective tissue diseases present in the same patient), chronic viral infection (hepatitis C or B virus or human immunodeficiency virus), or acute bacterial, viral, or fungal infections were excluded from the study.

### Clinical Assessment and Laboratory Measurements

Disease activity and functional disability were evaluated through the Disease Activity Score 28 (DAS28) and a Spanish version of the Health Assessment Questionnaire (HAQ-DI) by a rheumatologist. The blood samples were collected after 8 h of overnight fast. The samples in EDTA tubes were used to evaluate hematologic parameters (Coulter AcT 5diff Hematologic Analyzer, Beckman Coulter, USA) and the erythrocyte sedimentation rate (ESR). Levels of C-reactive protein (CRP) and rheumatoid factor (RF) (Othoclinical Diagnostics, VITROS®5600, USA) were also assessed according to the manufacturer's instructions.

### Parasitological Assessment

The stool samples were collected from patients and placed into plastic vials without preservatives. The stool samples were microscopically analyzed for the detection of parasitic elements by directs fecal smears: saline and iodine wet-mount preparations and the concentration by sedimentation method according to the procedures recommended by Pan American Health Organization/World Health Organization (PAHO/WHO) (43) and Clinical and Laboratory Standards Institute (CLSI) (44), the abundance scale was defined according to quantitate protozoa as follows: Number/10 Oil Immersion Fields (1000×); scarce ≤2; moderate 3–9, and abundant, ≥10. The parasitic elements were identified using the atlas of human parasitology (45) and the bench aids for the diagnosis of intestinal parasites by PAHO/WHO (43).

### Coprological Analysis

The pH was measured following the technique described by Manzano and Nogueda (46). Briefly, the samples diluted 1:2 with physiological saline were centrifuged at 500×*g* for 10 min. The pH test strips, 0–14 pH (Merck®), were introduced into the supernatant and compared, as soon as possible, with the supplier's reference color table.

The presence of mucus, Charcot–Leyden crystals, fatty acids, soaps, and undigested foods (starches, meat, and vegetable fibers) was determined by observing fecal smears stained with Lugol. The presence of neutral fats was determined with the Sudan III stain (46, 47).

### Serum Quantification of IFABP2 and Cytokines (TNF-α, IL-10, and IL-17A)

The IFABP2 serum levels were determined using ELISA kits (Sigma-Aldrich, USA), following the manufacturer's instructions. The results were expressed as ng/ml, and the detection limit was 0.025 ng/ml. Similarly, TNF-α levels were quantified using ELISA kits (Invitrogen, Thermo Fisher Scientific, Vienna, Austria). IL-10 and IL-17A were quantified using a ProcartaPlex assay (Invitrogen, Thermo Fisher Scientific, Vienna, Austria), following the specifications from the manufacturers. Cytokine levels are expressed in pg/ml.

### Separation of PBMCs and Flow Cytometry

Peripheral blood mononuclear cells (PBMCs) were separated by Ficoll density gradient (Axis-Shield, Oslo, Norway) and cryopreserved in freezing medium (10% DMSO, 90% fetal calf serum; Lonza, CA, USA) in liquid nitrogen until used. PBMCs were thawed, washed twice with phosphate-buffered saline (PBS, Lonza, CA, USA), and stained at room temperature for 15 min with the following antibodies: BV570-CD3 (clone UCHT1), APC-Cy7-CD4 (clone A161A1), and Alexa Fluor 700-CD8 (clone RPA-T8) (BD Biosciences, San Jose, CA, USA). Viability was determined using live/dead aqua fluorescent reactive dye (Thermo Fisher Scientific, CA, USA). Cells were washed and fixed in 300 μl of 1% paraformaldehyde (Sigma-Aldrich, St. Louis, MO, USA) and acquired immediately in a FACS Symphony cytometer (BD, Biosciences). Fluorescence minus one stained tubes were used as gating controls. Data were analyzed using FlowJo v.10 software (FlowJo LLC, Ashland, OR, USA) (Supplementary Figure S1).

### Statistical Analysis

Statistical analysis was carried out using Stata v.14.0 (StataCorp, College Station, TX, USA) and GraphPad Prism v.8.0 (GraphPad Software, San Diego, CA, USA) for Windows. Categorical data were expressed as numbers and proportions, and they were compared using the Chi-squared test. Continuous variables were compared among groups by Mann–Whitney U Test. The *p* <0.05 indicated statistical significance.

## Results

### Demographic Data and Clinical Features According to Protozoa Infection Status

**Figure 1:**
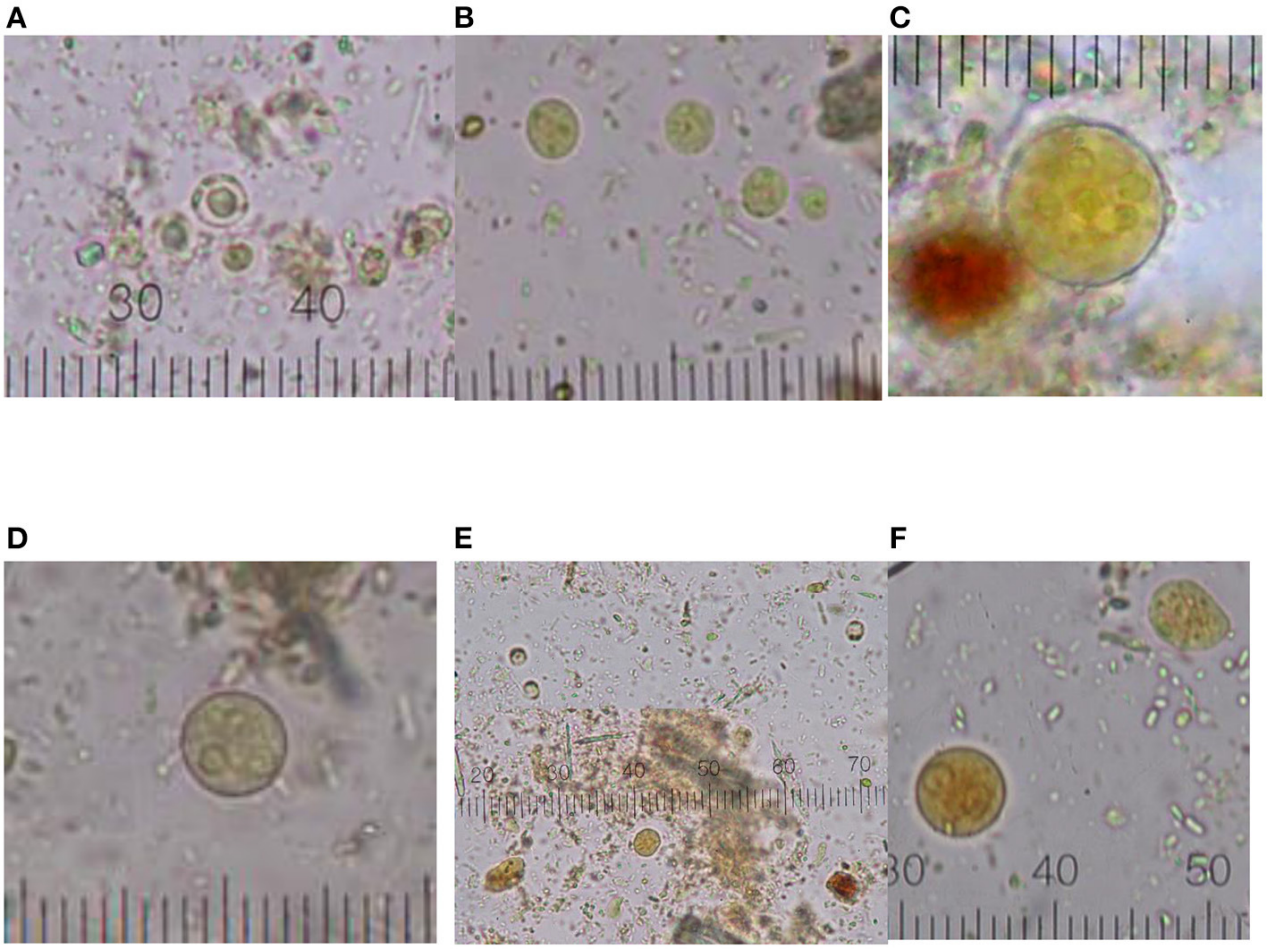
Graphical distribution of intestinal protozoa cysts. **(A)** *Blastocystis sp*.; **(B)** *End. nana*; **(C)** *Ent. coli*; **(D)** *Ent. hystolytica/dispar*; **(E)** coinfection (*Blastocystis* sp. and *End. nana*); **(F)** coinfection multiple (*Ent. hystolytica/dispar, End. nana, Blastocystis* sp.). Micrometric coefficient: 2.5 μm **(A,C–F)**; 1 μm **(B)**.

Eighteen women diagnosed with RA were included in this study. The median age of the participants was 46.5 years old. According to the DAS28-ESR score, the population presents moderate clinical activity. The 66.7% (*n* = 12) of the individuals had pharmacological prescriptions with disease-modifying antirheumatic drugs (DMARDs), and 33.3% (*n* = 6) were recently diagnosed and provided biological samples before the beginning of their prescribed treatment. When compared to the clinical characteristics and laboratory parameters according to the presence of protozoa, there were no significant differences identified (Table 1). We found that none of the fecal samples were positive for nematodes or platyhelminths; only protozoa were identified (Figure 1).

**Table 1:** Demographic, clinical, and inflammatory parameters according to the infection status by intestinal protozoa in patients with RA.

		**Protozoa infection status**		
**Variables**	**Total (*****n*** **= 18)**	**Negative (*****n*** **= 9)**	**Positive (*****n*** **= 9)**	***p*** **-value**
**Sociodemographic features**				
Age, years, median (P_5−_P_95_)^a^	46.5 (23–67)	47 (36–67)	46 (23–53)	0.16
Body mass index, kg/m^2^, median (P_5−_P_95_)^a^	28.1 (21.3–39.1)	28.1 (24.8–38.9)	28.1 (21.3–39.1)	0.85
Communities				0.06
Urban	8 (44.4)	3 (33.3)	7 (77.8)	
Rural	10 (55.6)	6 (66.7)	2 (22.2)	
**Clinical assessment**				
Disease evolution, years, median (P_5−_P_95_)^a^	3.49 (0.33–25)	3.98 (1–25)	2.73 (0.33–16)	0.89
CRP, mg/L, median (P_5−_P_95_)^a^	13.9 (4.99–46.9)	13.4 (7.5–46.9)	14.4 (4.9–33.9)	0.33
ESR, mm/hr, median (P_5−_P_95_)^a^	36 (7–51)	37 (7–50)	35 (12–51)	0.69
DAS28-ESR, score, median (P_5−_P_95_)^a^	3.22 (2.45–6.56)	3.39 (2.55–6.56)	2.91 (2.45–5.13)	0.23
DAS28-ESR, *n* (%)^b^				0.57
Remission (<2.6)	3 (16.7)	1 (11.1)	2 (22.2)	
Low activity (≥2.6, <3.2)	6 (33.3)	2 (22.2)	4 (44.4)	
Moderate activity (≥3.2, ≤ 5.1)	6 (33.3)	4 (44.4)	2 (22.2)	
High activity (>5.1)	3 (16.7)	2 (22.2)	1 (11.1)	
HAQ-DI, score, median (P_5−_P_95_)^a^	0.27 (0–1.15)	0.55 (0.05–1.15)	0.05 (0–0.8)	0.09
**Laboratory assessment**				
Rheumatoid factor, IU/ml, median (P_5−_P_95_)^a^	65.9 (8.6–942.5)	76.7 (8.56–942.5)	59.9 (8.59–443)	0.82
Rheumatoid factor, *n* (%)^b^				0.59
Negative (<20 UI/ml)	5 (27.8)	3 (33.3)	2 (22.2)	
Positive (≥20 UI/ml)	13 (72.2)	6 (66.7)	7 (77.8)	
Leucocytes, (%)^b^	7.18 (4.3–14.2)	6.9 (4.3–13.2)	8 (5.3–14.2)	0.10
Neutrophils, (%)^b^	62.5 (43–71)	62 (43–71)	63 (45–71)	0.92
Basophiles, (%)^b^	0.45 (0–1)	0.4 (0–0.6)	0.7 (0–1.0)	0.15
Monocytes, (%)^b^	5.8 (1–10.1)	6 (4–8.8)	5.6 (1–10.1)	0.56
Eosinophils, (%)^b^	2.9 (0–12)	2 (0–6)	3 (0.5–12.0)	0.16
Lymphocytes, (%)^b^	29 (18.3–48)	29 (18.3–48)	29.1 (21–48)	0.92
**Current therapy scheme**				
DMARDs, *n* (%)^b^				0.31
No	6 (33.3)	2 (22.2)	4 (44.4)	
Yes	12 (66.7)	7 (77.8)	5 (55.6)	

*DAS28, disease activity score 28; CRP, C-reactive protein; DMARDs, disease-modifying antirheumatic drugs; ESR, erythrocyte sedimentation rate; HAQ-DI, health assessment questionnaire; RA, rheumatoid arthritis*.

a *Data are expressed as the median and percentiles 5th−95th, compared using Mann–Whitney U test*.

b *Data are expressed as the n (%), compared using the Chi-squared test*.

*p < 0.05 was considered statistically significant*.

**Figure 2:**
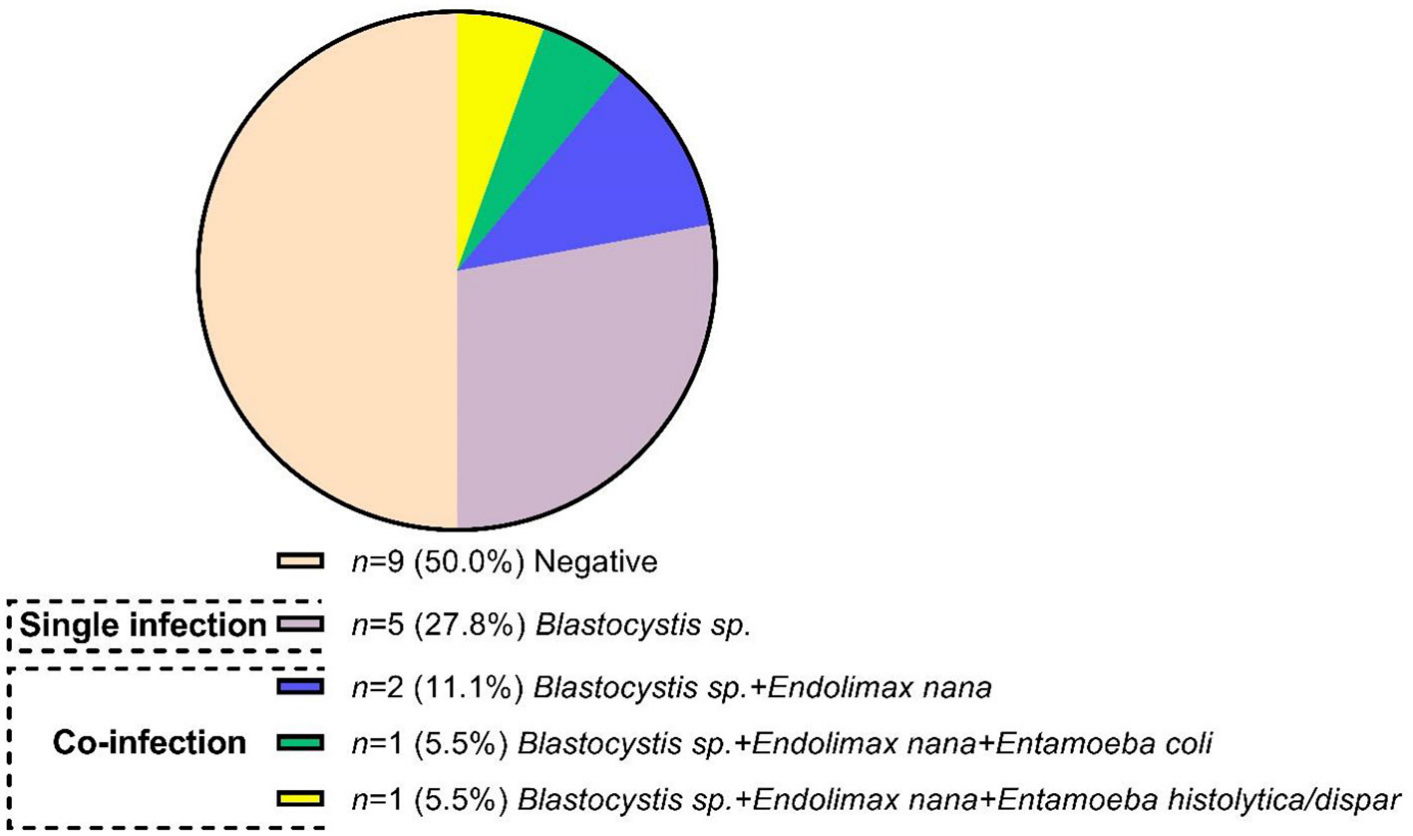
Frequency and infection protozoa status in patients with rheumatoid arthritis.

A total of 9 patients (50%) (four patients newly diagnosed and five under a therapeutic scheme with DMARDs) were positive for intestinal protozoa. Of those nine patients, 22.2% had a status of single infection with *Blastocystis sp*., and 27.8% had coinfection with *Blastocystis sp*. co-existing with one or more of the following protozoa: *End. nana, Entamoeba coli, Ent. histolytica*, or *Entamoeba dispar* (Figure 2).

Within the positive samples for *Blastocystis sp*., the abundance scale was distributed as follows: scarce on 18.2%, moderate on 9.1%, and abundant on 13.6%. For *End. nana*, the abundance scale was scarce on 4.5%, moderate on 4.5%, and abundant on 9.1% within the positive population, while for *Ent. coli* and *Ent. histolytica/Ent. dispar*, the identified scale was scarce on 4.5% of the positive population. On the other hand, the coprological analysis revealed higher quantities of neutral fat in patients who were positive for protozoa (*p* < 0.001), whereas starches, nondigestible fibers, and fatty-acid crystals were present similar between groups. The characteristics of the fecal analysis of each patient that were positive for protozoa are summarized in Supplementary Table S1.

### Association Between IFABP2 Serum Levels in Infection Status in RA

**Figure 3:**
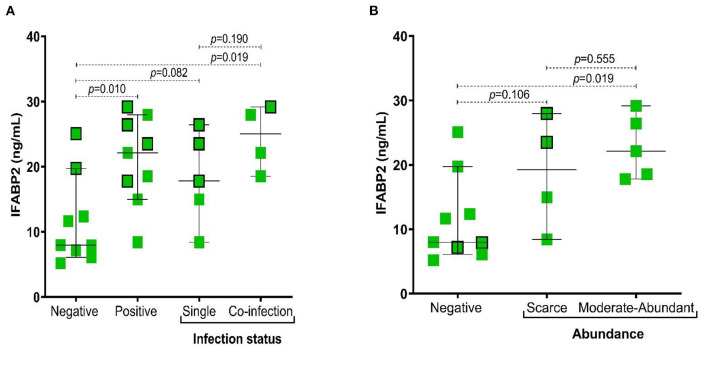
Association of IFABP2 levels according to infection status by intestinal protozoa and abundance of *Blastocystis sp*. **(A)** IFABP2 serum levels according to infection status; **(B)** IFABP2 serum levels according to the abundance of *Blastocystis sp*. Bars represent the median with the P_5_-P_95_ range. The boxes with borders represent patients without pharmacological therapy. Statistical analyses were performed by Mann–Whitney U test. Significance was set at *p* < 0.05.

In the present study, we observed the IFABP2 serum levels were significantly higher in patients positive for infection with intestinal protozoa (*p* = 0.010), particularly in those with coinfection (*p* = 0.019) (Figure 3A) and with a higher abundance of *Blastocystis sp*. (*p* = 0.019) (Figure 3B).

**Figure 4:**
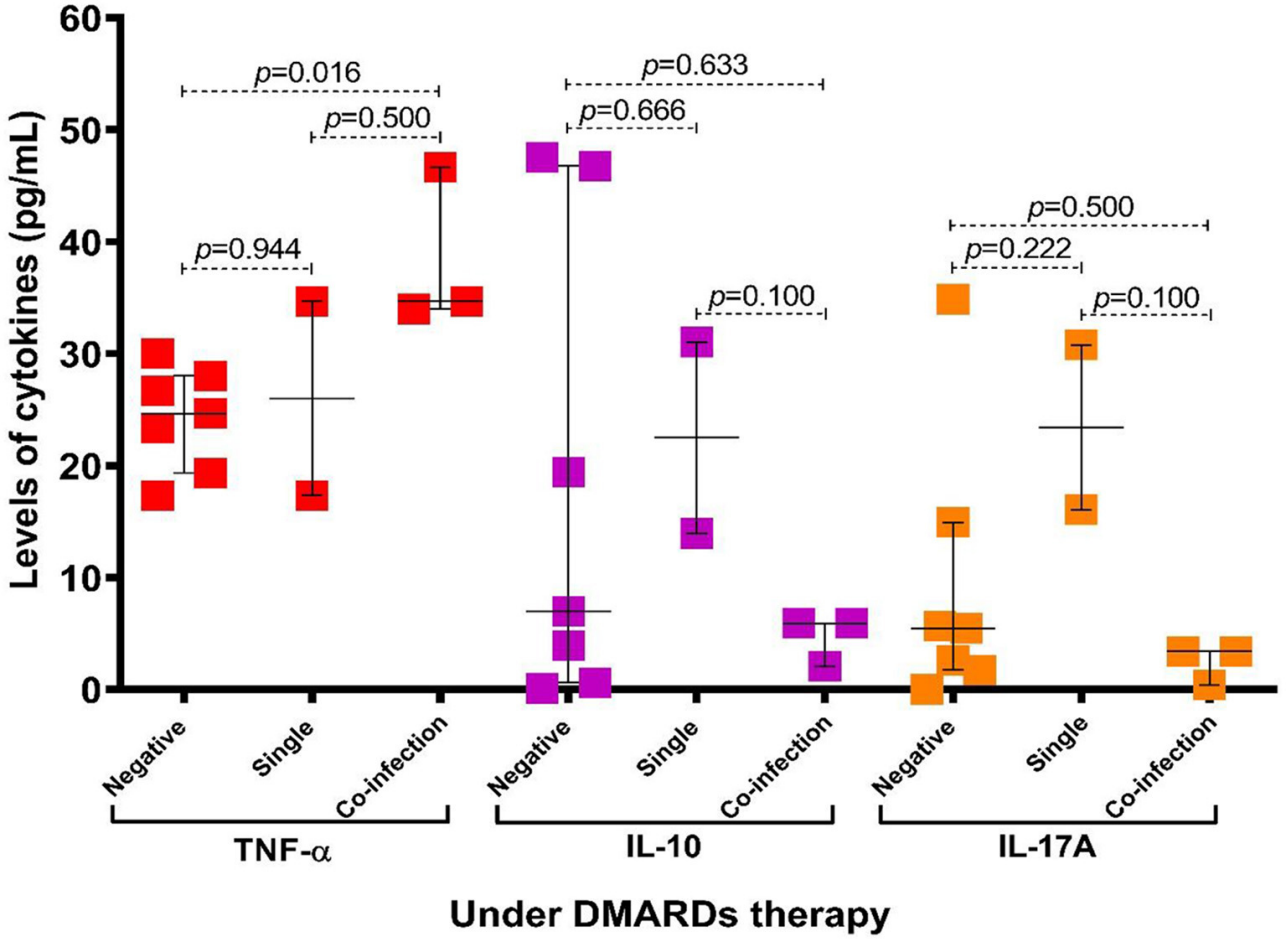
Association of levels of cytokines according to infection status by intestinal protozoa and patients with RA under DMARDs therapy. Bars represent the median with P_5_-P_95_ range. Statistical analyses were performed by Mann–Whitney U test. Significance was set at *p* < 0.05.

**Figure 5:**
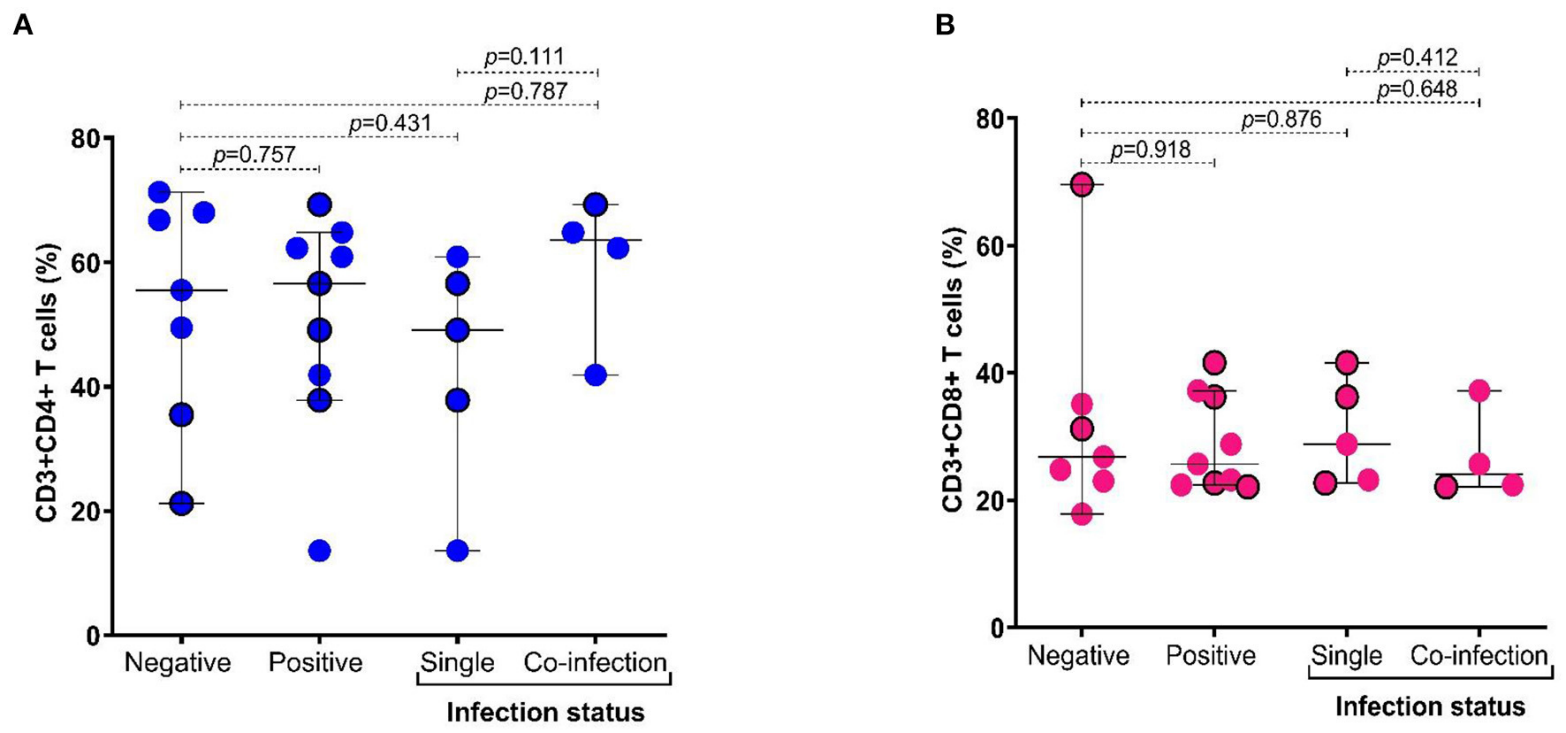
Distribution of frequency of CD4+ and CD8+ T cells according to infection status by intestinal protozoa. **(A)** Frequency of CD3+ and CD4+ T cells (%); **(B)** frequency of CD3+ and CD8+ T cells (%). Bars represent the median with the P_5_-P_95_ range. The circles with borders represent patients without pharmacological therapy. Statistical analyses were performed by Mann–Whitney U test. Significance was set at *p* < 0.05.

When the cytokines' levels were compared between groups of patients according to therapy and protozoa infection status, we found that the levels of TNF-α were higher in patients with coinfection (*p* = 0.016), particularly in the group of patients under antirheumatic treatment (Figure 4). Additionally, we observed that in subjects with coinfection with intestinal protozoa, the levels of TNF-α (Supplementary Figure S2) and the frequency of CD4+ T cells were higher in participants with coinfection by protozoa, although not significantly (Figure 5A), while CD8+ T cells were similar between groups (Figure 5B).

## Discussion

The main findings in this study show that (i) infection by *Blastocystis sp*. is frequent in patients with RA in southern Mexico, (ii) serum levels of IFABP2 were higher in individuals with infection and coinfection of protozoa, (iii) the abundance of *Blastocystis sp*. is associated with higher serum levels of IFABP2, and (iv) infection by protozoa promotes the increase of TNF-α in patients with RA, mostly in those under DMARDs pharmacological treatment.

The prevalence of infection by intestinal protozoa in subjects with rheumatic diseases has been previously reported to be particularly high (25–30%) (48), when compared to that reported in Mexican population (49) and asymptomatic (50). In the present study, we found that 50% (9/18) of the individuals with RA recruited were positive for intestinal protozoa, similarly reported in an Egyptian population with RA (51), and higher than the report of Jimenez-Balderas et al. (48) for patients with RA in Mexico City [25%, (3/12)], and also the one reported for patients with RA in Brazil [11.9%, (8/67)] (20). These differences could be explained by sociodemographic factors that influence risk of parasitic infection, such as living in rural areas, lacking basic services (drinking water, for example), and the number of inhabitants per household (52), poor personal hygiene (50), and living with pets (48, 53). Another important factor for increased susceptibility for protozoa coinfection in patients with RA could be caused by pharmacological DMARDs treatment.

In our study, the number of positive cases for protozoa infections among patients with or without treatment did not show significant differences. However, patients under DMARDs treatment presented a higher frequency of coinfection (4/5) when compared to patients without treatment (1/4). Treatment with conventional DMARDs has been associated with the development of severe and moderate parasitic infections in RA (54).

Parasite's infection by *Microsporidio* (18), *Endo. nana* (20), and *Cryptosporidium* (51) has been previously reported in patients with RA. Hussein et al. (51) reported up to 46.72% (50/107) of positive cases of parasitic infection in the Egyptian population, identifying mainly the presence of *Cryptosporidium* (48%), *Cyclospora cayetanensis* (32%), *Giardia lamblia* (24%), *Blastocystis hominis* (20%), and *Ent. histolytica* (8%). In addition, Jimenez-Balderas et al. (48) reported single infections by *Ent. coli* and *B. hominis*, and one case of mixed infection by *B. hominis* and *Ent. histolytica* in Mexican patients. In the present study, we also found infection by *End. nana*. Also, we observed that the frequency of coinfection was 6 times higher than in others reports in the asymptomatic population, in which a percentage of 2% (50) and 3.4% of coinfection was identified (55).

Reactivation of RA has been associated with infection by intestinal parasites, and it is suggested that parasitic coinfection could compromise the immune response of susceptible hosts (16). Additionally, it has been reported that parasitic infection is accompanied by joint pain and elevated levels of ESR and CRP (51). In patients with RA, infection with *End. nana, Ent. histolytica*, and *Ent. coli* has been related to poor health scales and fatigue (20), but not with the parameters of disease's clinical activity (18), similar to what was found in this study. Nevertheless, *B. hominis* has been particularly associated with reactive RA in four clinical cases (13, 17, 19). Our study found infection by *Blastocystis sp*. in every positive case. We found that *Blastocystis sp*. abundance and its coexistence with other intestinal protozoa were associated with increase of IFABP2 and TNF-α serum levels, thus suggesting a relation between infection by intestinal protozoa and the potential damage of the intestinal epithelium and inflammation in patients with RA.

In this sense, it has been reported that *B. hominis* increases intestinal permeability by promoting the damage of epithelial cells through apoptosis and the degradation of tight junctions such as occludin and ZO-1 by two cysteine proteases (legumain and capthesin B). Furthermore, *B. hominis* has an immunomodulatory effect on the degradation of IgA, the inhibition of iNOS, and the increase of cytokines such as IL-8, GM-CSF, IL-1β, IL-6, and TNF-α (32, 56), and this could explains the increase of TNF-α in our patients with coinfection with *Blastocystis sp*. Also, it has been described that coinfection by *Blastocystis sp*. and *Ent. histolytica* modulates the apoptosis of intestinal epithelial cells (27); therefore, the coinfection status could strengthen the intestinal lesion. In our study, one case of coinfection by *Blastocystis sp., Ent. histolytica*, and *End. nana* was identified, and it was observed that even though it was in a patient under DMARDs triple-therapy treatment, the patient presented moderate clinical activity (DAS28-ESR = 3.07), a higher proportion of eosinophils (9%), and the presence of Charcot–Leyden crystals.

This study establishes a relation between the infection by *Blastocystis sp.*, its abundance, and the serum levels of IFABP2. Previously, it has been shown that increased intestinal permeability happens during the infection by intestinal protozoa, and that the damage of the intestinal epithelium is enhanced during mixed infection by *G. lamblia* and *B. hominis* (57). It has been proved that subtype 7 of *B. hominis* promotes the apoptosis of enterocytes *in vitro* by activating the caspases 3 and 9 (intrinsic pathway) (31). Particularly, capthesin B virulence factor of subtype 7 increases the intestinal permeability *in vitro* (58), and it promotes *in vivo* the decrease of *Bifidobacterium* and *Lactobacillus* (10). Even though we did not investigate the *Blastocystis* subtypes, it is important to identify the presence of subtypes with higher virulence and its effects on the intestinal barrier (59), the gut microbiota, and the variability of the clinical phenotype of RA disease.

We observed in this study that the serum levels of TNF-α and the frequency of CD4+ T cells in peripheral blood were higher in patients with coinfection with protozoa; particularly in patients under DMARDs therapy and coinfection with protozoa, the TNF-α levels were significantly higher. The TNF-α serum levels are associated with prognostic response to DMARDs (60). Therefore, the presence of elevated levels of TNF-α and the increase of lymphocytes T CD4+ in patients under DMARDs treatment could suggest a relation between the poor response to treatment and the infection by protozoa, even though more robust studies are required to delve into this aspect.

We demonstrated the relation between the infection by protozoa and intestinal permeability, and potentially with inflammation in patients with RA. Nevertheless, our study presents some limitations worthy of consideration: (1) the design of the study was cross-sectional, and only one fecal sample of each participant was analyzed, (2) the sample size was small, and (3) individuals with and without treatment were included, which could represent a limitation for proving the relationship between the presence of intestinal protozoa and inflammation markers.

In conclusion, *Blastocystis sp*. was the most common intestinal protozoa found in women with RA from southern Mexico. Elevated levels of IFABP2 were found in individuals with coinfection by intestinal protozoa, mainly, in whom the abundance of *Blastocystis sp*. was higher. The coinfection with protozoa was more frequent in patients under DMARDs treatment, associated with higher levels of serum TNF-α.

Therefore, our results suggest that the infection with protozoa is associated with intestinal barrier damage and with the modulation of pro-inflammatory conditions in patients with RA; thus, our findings evidence the need to conduct a screening for intestinal parasites in patients with RA mainly during the first year of antirheumatic treatment to prevent epithelial barrier damage and side effects associated with pro-inflammatory status.

## Data Availability Statement

The original contributions presented in the study are included in the article/Supplementary Material, further inquiries can be directed to the corresponding author.

## Ethics Statement

The studies involving human participants were reviewed and approved by Ethics and Research Committee of the University Autonomous of Guerrero (Project identification code: CB-004/2017). The patients/participants provided their written informed consent to participate in this study.

## Author Contributions

IG-G: conceptualization and design of the study and drafted the final manuscript. OZ-G and IG-P: data collection and population sampling. BN-T: performed the stool analysis. JN-Z: performed the clinical evaluation of patients. OB: performed the determination and analysis of T cells. OZ-G, RF-V, GP-R, IG-P, and IP-R: performed the proteins' determination. IG-G and OZ-G: statistical analysis. All authors contributed to manuscript revision, read, and approved the submitted version.

## Conflict of Interest

The authors declare that the research was conducted in the absence of any commercial or financial relationships that could be construed as a potential conflict of interest.

## Publisher's Note

All claims expressed in this article are solely those of the authors and do not necessarily represent those of their affiliated organizations, or those of the publisher, the editors and the reviewers. Any product that may be evaluated in this article, or claim that may be made by its manufacturer, is not guaranteed or endorsed by the publisher.
