# Supplementary material for: The Infection, Coinfection, and Abundance of Intestinal Protozoa Increase the Serum Levels of IFABP2 and TNF-α in Patients With Rheumatoid Arthritis
Source: Front Med (Lausanne). 2022 Apr 12;9:846934. doi: 10.3389/fmed.2022.846934 (PMC9039364; doi:10.3389/fmed.2022.846934)
Supplement: Supplementary file 1 [file Data_Sheet_1.pdf]

Supplementary Material

Supplementary Table 1. General characteristics according to individual positive RA patients

Variables	Case 1	Case 2	Case 3	Case 4	Case 5	Case 6	Case 7	Case 8	Case 9
**Sociodemographic data**									
Age	47	33	51	53	23	53	46	43	27
Locality	Rural	Urban	Rural	Rural	Rural	Rural	Rural	Urban	Rural
**Current therapy scheme**	Newly diagnosed	Newly diagnosed	Newly diagnosed	Newly diagnosed	MTX	MTX	MTX+PDN	MTX	MTX+CLQ+SSZ+PDN
**Stool analysis**									
pH (units)	8	5	5	7	7	6	7	6	7
Neutral fats	Scarce	Scarce	Scarce	Scarce	Scarce	Moderate	Scarce	Moderate	Absent
Mucus	Absent	Scarce	Scarce	Absent	Absent	Absent	Absent	Absent	Absent
Food undigested	Scarce	Moderate	Scarce	Increase	Scarce	Moderate	Scarce	Scarce	Scarce
Starches	Scarce	Scarce	Absent	Moderate	Scarce	Scarce	Scarce	Moderate	Moderate
Vegetable fibers undigested	Scarce	Absent	Scarce	Scarce	Scarce	Scarce	Absent	Moderate	Scarce
Fatty acids crystals	Increase	Scarce	Scarce	Increase	Increase	Increase	Increase	Increase	Increase
Charcot Leyden crystals	Absent	Absent	Absent	Absent	Absent	Absent	Absent	Absent	Scarce
Protozoa Infection	*Blastocystis sp.*	*Blastocystis sp.*	*Blastocystis sp.*	*Blastocystis sp.; Endolimax nana; Entamoeba coli*	*Blastocystis sp.*	*Blastocystis sp.*	*Blastocystis sp.; Endolimax nana*	*Blastocystis sp.; Endolimax nana*	*Blastocystis sp.; Endolimax nana; Entamoeba coli; Entamoeba histolytica/dispar*
Parasite abundance	Scarce	Scarce	Moderate	Abundant; abundant; scarce	Scarce	Moderate	Scarce; scarce	Abundant; moderate	Abundant; abundant; scarce; scarce
Status Infection	Single	Single	Single	Co-infection	Single	Single	Co-infection	Co-infection	Co-infection

CLQ, chloroquine; MTX, methotrexate; *PDN*, prednisone; *RA*, rheumatoid arthritis; *SSZ*, sulphasalazine.

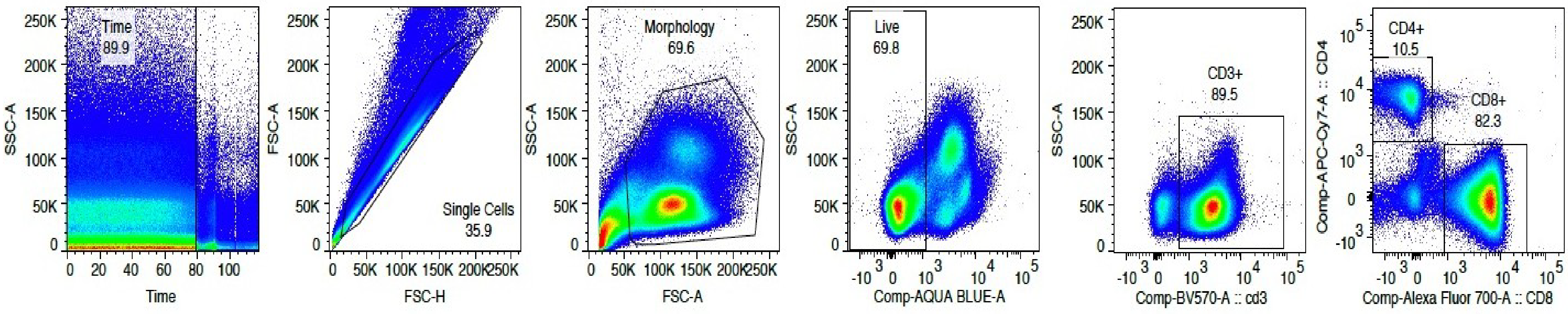

Supplementary Figure 1 | Gating strategy for cytometry assay for CD4+ and CD8+ T cells. The figure shows the gating strategy for the analysis of CD3 and CD4+ T cells and all samples. First define singles and morphology by using forward versus side scatter, followed by the exclusion of dead cells (aqua dye negative events). Live cells were gated on CD3+ and CD4+ or CD8+ T cells.

A) B) B)

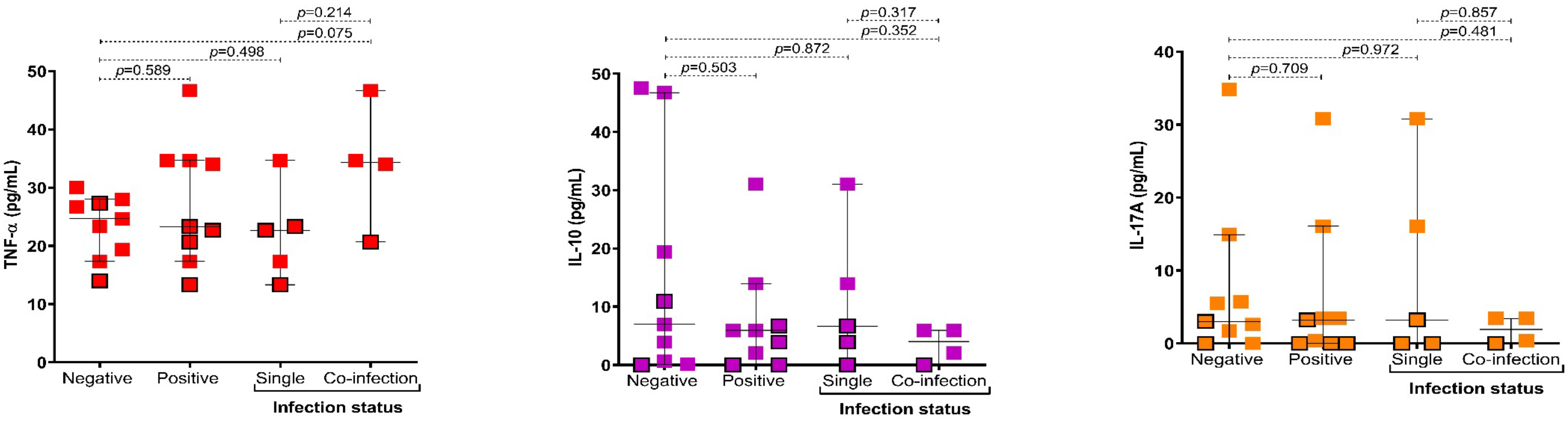

Supplementary Figure 2 | Association between cytokines levels according to status infection by intestinal protozoa. A) TNF-α serum levels; B) IL-10 serum levels; C) IL-17A serum levels. Bars represent the median with the P_5-P_{95} range. The boxes with borders represent patients without pharmacological therapy. Statistical analyses were performed by Mann-Whitney U test. Significance was set at p-value <0.05.
